# Spatial and Temporal Analysis of Lung Cancer in Shenzhen, 2008–2018

**DOI:** 10.3390/ijerph18010026

**Published:** 2020-12-22

**Authors:** Lin Lei, Anyan Huang, Weicong Cai, Ling Liang, Yirong Wang, Fangjiang Liu, Ji Peng

**Affiliations:** 1 Department of Cancer Control and Prevention, Shenzhen Center for Chronic Disease Control, Shenzhen 518020, China; lin.leilana@gmail.com (L.L.); 19ayhuang@stu.edu.cn (A.H.); 16wccai@alumni.stu.edu.cn (W.C.); liangling85@163.com (L.L.); yrwang1991@126.com (Y.W.); liufangjiang1994@163.com (F.L.); 2 Mental Health Center, Shantou University Medical College, North Taishan Road, Shantou 515065, China

**Keywords:** cancer registry, incidence rate, lung cancer, spatial autocorrelation, temporal trend

## Abstract

Lung cancer is the most commonly diagnosed cancer in China. The incidence trend and geographical distribution of lung cancer in southern China have not been reported. The present study explored the temporal trend and spatial distribution of lung cancer incidence in Shenzhen from 2008 to 2018. The lung cancer incidence data were obtained from the registered population in the Shenzhen Cancer Registry System between 2008 and 2018. The standardized incidence rates of lung cancer were analyzed by using the joinpoint regression model. The Moran’s I method was used for spatial autocorrelation analysis and to further draw a spatial cluster map in Shenzhen. From 2008 to 2018, the average crude incidence rate of lung cancer was 27.1 (1/100,000), with an annual percentage change of 2.7% (*p* < 0.05). The largest average proportion of histological type of lung cancer was determined as adenocarcinoma (69.1%), and an increasing trend was observed in females, with an average annual percentage change of 14.7%. The spatial autocorrelation analysis indicated some sites in Shenzhen as a high incidence rate spatial clustering area. Understanding the incidence patterns of lung cancer is useful for monitoring and prevention.

## 1. Introduction

Lung cancer is one of the most common cancers in the world, which brings a heavy burden to people’s health and social economy. With the acceleration of industrialization, urbanization and aging of the population, the harm of lung cancer has become increasingly prominent [1]. A cancer statistics research across 20 regions of the world showed that lung cancer accounted for the largest proportion of total new cancer cases (11.6%) and far more deaths than other cancers in 2018 [2]. As the same trend in the world, lung cancer is also the most common cancer and the leading causes of cancer-related death in China [3]. From 1990 to 2017, the age-standardized Year of Life Loses (YLLs) and Disability Adjusted of Life Years (DALYs) due to lung cancer increased by 12.6% (1/100,000) and 13.1% (1/100,000), respectively, which shows that the burden brought by lung cancer is increasing gradually and cannot be ignored [4].

There are three main histological classifications of lung cancer, adenocarcinoma (ADC), squamous cell carcinoma (SQCC) and small cell carcinoma (SMCC), accounting for about 40%, 25–30% and 10–15% of all lung cancers, respectively [5]. ADC and SQCC both belong to non-small cell lung cancer, among which, ADC mainly occurs in the peripheral bronchi, while SQCC mainly occurs in the main bronchi. The SMCC of the lung is an invasive tumor of neuroendocrine origin with a high degree of malignancy, strong aggressiveness and almost no bronchial invasion. Although the incidence of SMCC has tended to decline, it remains one of the important causes of lung cancer death. In recent years, many studies have reported the temporal variation trend of lung cancer by sex and histological type. According to the statistics from the World Health Organization, ADC has overtaken SQCC as the most common type of lung cancer in many countries [6]. In China, similar results were reported in several studies of temporal trends in lung cancer histological types. The studies in Beijing, Tianjin, and Sichuan all reported an increasing trend in ADC [7,8,9]. Although the incidence of SQCC was decreasing, SQCC remained the most common histologic type of lung cancer in males in Tianjin [8]. They suggest that the decline in smoking prevalence may partly explain the change in long-term trends in SQCC incidence and histological types. In foreign countries, the incidence trend of different histological types of lung cancer was noticed earlier than in China. A Japanese study reported a decreasing rate in SQCC and SMCC, and an increasing rate in ADC [10]. Similarly, an increasing incidence of ADC in lung cancer in both males and females was observed in Estonia [11]. This trend may be related to the difference of historical patterns of tobacco use or to modern filtered cigarette smoke [12]. Especially, with the advent of e-cigarettes, the variation about incidence trend and histological types of lung cancer will become more complex. Some studies have confirmed that exposure to e-cigarettes can induce lung adenocarcinoma, induce DNA damage in the lungs, and inhibits DNA repair in lung tissues in mice [13,14]. Moreover, an aging population and improved diagnostic techniques are likely to lead to an increase in lung cancer rates. However, so far, there are still few studies on the incidence trend of lung cancer by histological type in China, especially in southern China. On the other hand, the spatial distribution of lung cancer incidence has become a hot topic. A study from Saudi Arabia using spatial autocorrelation analysis revealed a statistically significant positive correlation in male lung cancer indicated a clustering pattern [15]. They believed that the aggregation of lung cancer in eastern regions may be partly due to the high prevalence of smoking. Moreover, other environmental factors are also important. An increase in lung cancer rates was linked to PM2.5 and ozone pollution, according to a study that combined lung cancer incidence with air pollution data from different regions [16]. Notably, different regions have obvious spatial heterogeneity in terms of climatic conditions, economic structure and industrial structure, resulting in different environmental pollution conditions. To explore the aggregation of cancer incidence in a certain area is helpful to analyze the relationship between cancer and various risk factors, and to carry out targeted prevention.

Therefore, we performed a spatial and temporal analysis to explore the incidence patterns of lung cancer in Shenzhen, using new lung cancer cases from the Shenzhen Cancer Registry System (SCRS) from 2008 to 2018. The authenticity of the population data included in the study was high due to the strict reporting process in SCRS. The present study can provide scientific basis for the prevention and control of lung cancer in Shenzhen.

## 2. Materials and Methods

### 2.1. Data Sources

The lung cancer cases from 2008 to 2018 were obtained from SCRS, which was established in 1998. The SCRS covered a total of 89 hospitals which were qualified for cancer diagnosis. These hospitals were required to regularly report new cancer cases using a unified cancer reporting card according to the International Classification of Diseases, 10th revision (ICD-10). Moreover, these data were supplemented by the Shenzhen Death Registration System to account for potentially under-reported cases. As Shenzhen is an immigrant city with large population mobility, we only included the registered population, who were coded as C33–C34 according to ICD-10 in the SCRS. Base on the criteria of the International Classification of Diseases for Oncology, Third Edition (ICD-O-3) [17], we divided the histological classification of lung cancer into four categories as follows: ADC (8140, 8141, 8200, 8211, 8230, 8250–8255, 8260, 8310, 8323, 8440, 8470, 8480, 8490, 8550), SQCC (8050, 8052, 8070–8076, 8083–8084), SMCC (8041–8045), other specified histological type (OST) (8012–8014, 8022, 8031–8033, 8082, 8123, 8240, 8246, 8249, 8430, 8560, 8562, 8720, 8800, 8810, 8811, 8830, 8840, 8972, 8973, 8980, 8990, 9040, 9041, 9043, 9080, 9120, 9133, 9140). The registered population data over the study period were released by Shenzhen Statistics Bureau. We calculated the age distribution of the population from 2008 to 2018 based on the annual population data registered in Shenzhen and two available population age distributions in 2000 and 2010 released by the Shenzhen Statistics Bureau.

### 2.2. Quality Control

The SCRS has a repeat check function, which can automatically screen out suspected repetitive cases. These suspicious report cards will be deleted if they are determined to be duplicates by staff. At the same time, the specially-assigned staff in the hospital used the IARCcrg Tools program provided by the International Agency for Research on Cancer (IARC) to complete the review of monitoring data, including gender, age, code and diagnostic basis. Moreover, in order to make up the missed cases in time and ensure the accuracy and completeness of the data, all of the qualified hospitals were required to conduct monthly cancer underreporting surveys and to evaluate the quality of the report cards.

### 2.3. Statistical Analysis

The Chinese standardized incidence rate (CSR) was calculated according to the Chinese standard population in 2000, and world standardized incidence rate (WSR) was calculated according to the Segi’s world standard population in 1982. The difference of lung cancer composition ratio in different years was calculated by chi-square analysis. The temporal trend of lung cancer was evaluated via the annual percentage change (APC) and the average annual percentage change (AAPC) using the joinpoint regression model [18]. APC is used to evaluate the internal trend of each independent interval of the piecewise function, or the global trend with the number of join points being zero. AAPC is used to comprehensively evaluate the global average change trend involving multiple regions. Since the distribution characteristics of lung cancer incidence are subject to Poisson distribution, we chose a log-linear model for calculation. The maximum number of two joinpoints was set for each analysis due to the short study period. Moreover, we used global spatial autocorrelation index Moran’s I to estimate the spatial clustering model of the total region, with its significant test [19]. The Moran’s I ranges from −1 to 1 and positive values represent the positive spatial correlation, which means that there is a strong aggregation among the regions; while negative ones indicate a negative spatial correlation among the regions. In other words, it indicated that high-incidence and low-incidence values are scattered. A Moran’s I equal to 0 represents a random pattern. If the overall space has a significant clustering pattern, we used local Moran’s I (Local Indicators of Spatial Association, LISA) to test the clustering type and the exact location. In addition, LISA map was developed to visualize the clustering of lung cancer incidence in Shenzhen. The aggregation areas with high and low incidence were marked in red and blue, respectively. The High–Low area which was marked in pink represents a higher incidence than the surrounding area, and the Low–High area marked in purple means that the incidence is lower in that area than in the surrounding area. The significant level was set to 0.05. The joinpoint analysis was used the Joinpoint Trend Analysis Software 4.8.1 (SEER*Stat, Bethesda, MD, USA). The ESRI ArcGIS software (U.S. Environmental Systems Institute, California, USA) was used to plot the spatial distribution of incidence rate of lung cancer cases during 2008 and 2018. The map of China was obtained from the Ministry of Natural Resources of the People’s Republic of China. Spatial cluster analysis was performed using the Geoda 1.14 software (GeoDa Center, Tempe, AZ, USA). The geographical level used in the maps of Shenzhen were subdistrict. MS Excel 2007 (Microsoft, Redmond, WA, USA) and Stata13 (Stat Corporation, College Station, TX, USA) were used for statistical analysis.

## 3. Results

### 3.1. Distribution and Incidence

The total and sex-specific incidence rates of lung cancer in Shenzhen from 2008 to 2018 are reported in Table 1. During the study period, a total of 9273 lung cancer cases were reported in Shenzhen, with the total crude incidence rate (CR) of 27.1 (1/100,000), CSR of 37.4 (1/100,000) and WSR of 37.5 (1/100,000). There were 5704 cases in males and 3569 cases in females, with a higher CSR in males (48.0/100,000) than in females (27.7/100,000).

**Figure 1:**
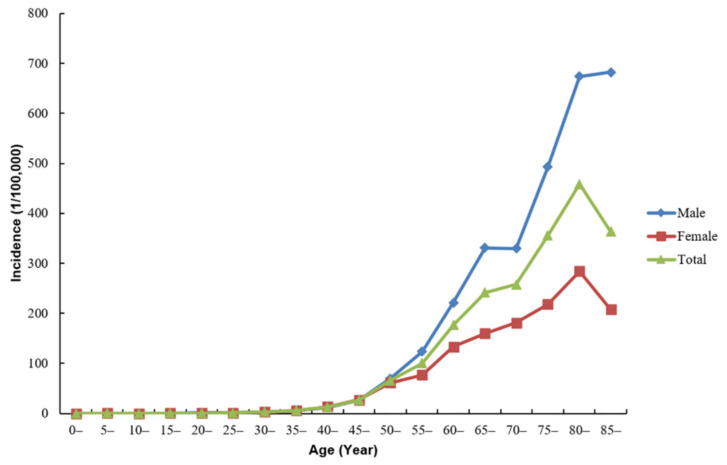
The age-specific incidence of new lung cancer cases in Shenzhen, 2008–2018.

Among all of the age groups, lung cancer was less common in people under 30 years old (4.8%) and more common from 40 to 70 years old (83.7%). During 2008 to 2018, the mean age at diagnosis of total lung cancer cases in Shenzhen was 62.9 years old. The age-specific incidence rate was relatively low until the age of 40, and had since increased sharply. The incidence rate in females peaked in the age group of 80–84 years, and then began to decline. By comparison, the incidence rate in males reached a peak after 85 years (Figure 1).

**Figure 2:**
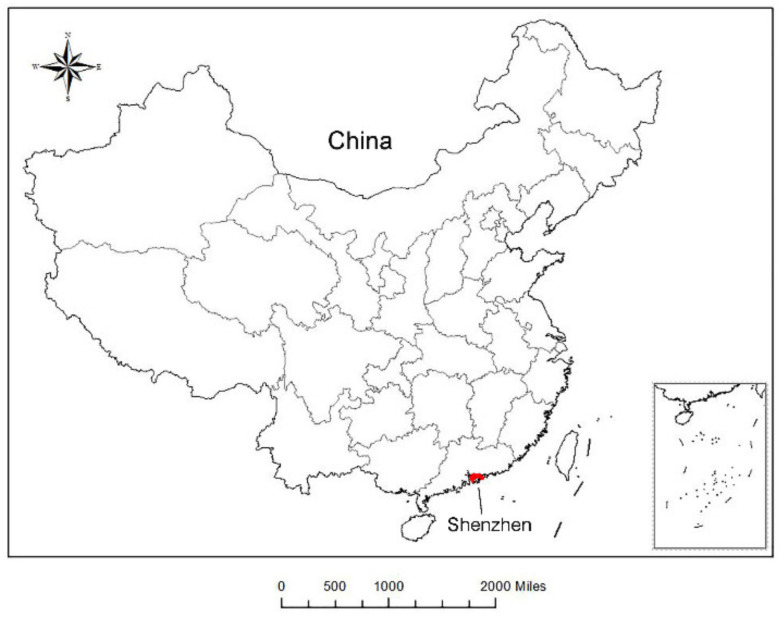
The geographical distribution of Shenzhen in China.

**Figure 3:**
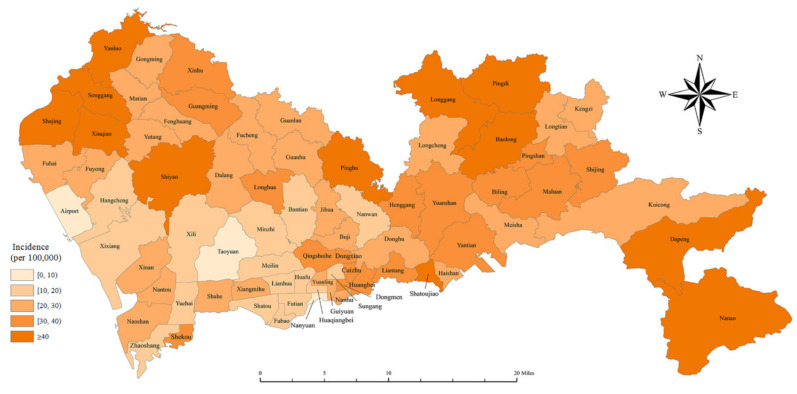
Geographic distribution of crude incidence of lung cancer in Shenzhen, 2008–2018. (Note: geographical level = subdistrict).

Shenzhen is in the southern part of China and belongs to the Pearl River Delta (Figure 2). The geographic distribution of the crude incidence rate of lung cancer in Shenzhen is shown in Figure 3, with the lowest incidence in the Taoyuan Subdistrict (CR: 8.9/100,000) in the Futian District and the highest incidence in the Dapeng Subdistrict (CR: 63.0/100,000) in the Dapeng District. In general, the incidence of lung cancer in rural areas was higher than in urban areas.

### 3.2. Histological Type

The distribution of lung cancer by histological type is shown in Table 2. Among the lung cancer cases reported in Shenzhen between 2008 and 2018, a total of 5648 (60.9%) cases were histologically diagnosed, up from 43% in 2008 to 72% in 2018 (χ^2^= 142.55, *p* < 0.001). The major histological types were ADC (69.1%), SQCC (15.0%) and SMCC (6.8%). From 2008 to 2018, the proportion of ADC gradually increased, especially in females. The proportion of ADC in females increased from 68.0% in 2008 to 89.6% in 2018 (χ^2^ = 13.73, *p* < 0.001), showing a relative increase of 31.8%. Moreover, the histological type distribution of lung cancer was different by gender. The proportion of SQCC and SMCC in males was higher than that in females (χ^2^ = 183.05, χ^2^ = 117.48, all *p* < 0.001), with the proportion of ADC in females was significantly higher than that in males (χ^2^ = 361.19, *p* < 0.001).

### 3.3. Trend Analysis

**Figure 4:**
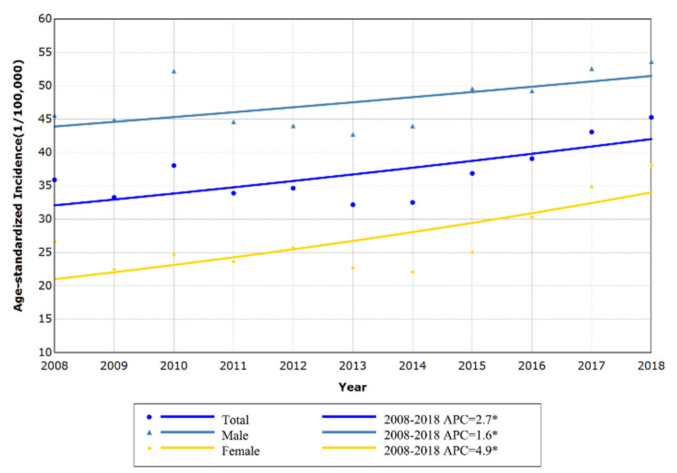
The trend of age-standardized incidence of lung cancer in Shenzhen, 2008–2018.

Based on the Chinese standard population in 2000, the APC of the age-standardized incidence rate of lung cancer by sex was calculated. The CSR of lung cancer in Shenzhen showed an increasing trend (APC: 2.7%; *p* = 0.011) between 2008 and 2018. Among them, the CSR in females (APC: 4.9%; *p* = 0.005) increased slightly faster than males (APC: 1.6%; *p* = 0.044) (Figure 4).

In three major histological types, the CSR of ADC in males had an increasing trend during 2008 to 2018, with an APC of 10.1% (*p* < 0.05). Among females, the overall trend of ADC was the same as males, but the incidence rate increased faster than males over the entire study period, with an APC of 14.7% (*p* < 0.05). However, the CSR of SQCC was different by sex. Among males, the incidence rate consistently showed a stable trend between 2008 and 2018 (APC: 1.1%, *p* > 0.05). In contrast, the incidence of SQCC in females showed a steady decreasing trend (APC: −5.4%, *p* < 0.05). Additionally, a stabilized trend of SMCC was observed both in males (APC: 2.6%, *p* > 0.05) and females (APC: −2.7%, *p* > 0.05). In other specified histological types of lung cancer, the CSR presented a steady trend in both males and females, with no statistical significance (*p* > 0.05) (Table 3).

### 3.4. Spatial Autocorrelation

The global spatial autocorrelation analysis of the cumulative CR of lung cancer in Shenzhen between 2008 and 2018 showed a Moran’s I index of 0.387 (z = 5.5, *p* < 0.01). It suggested that the incidence of lung cancer in Shenzhen had a certain degree of spatial clustering.

**Figure 5:**
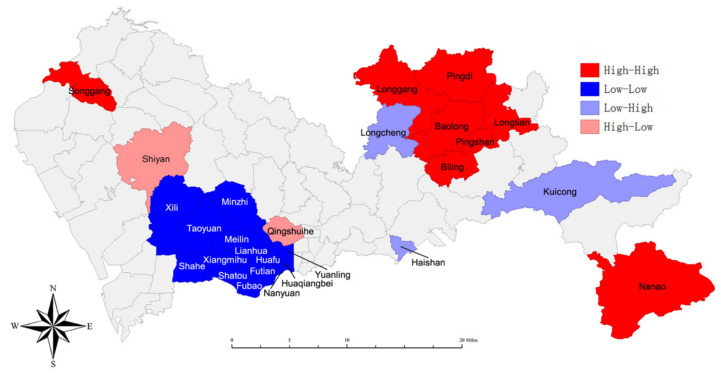
Local hotspot map for crude incidence of lung cancer in Shenzhen, 2008–2018. (Note: high–high = areas with high incidence; low–low = areas with low incidence; low–high = a low incidence area surrounded by high incidence areas; high–low = a high incidence area surrounded by low incidence areas).

We further made a LISA cluster map (Figure 5) to show the spatial clustering of local areas. The LISA visualization analysis demonstrated the presence of a hotspot (high–high) of lung cancer incidence in the Songgang Subdistrict of the Luohu District, the Nanao Subdistrict of the Dapeng District and several subdistricts of Pingshan and Longgang District. The low–low region represented by blue belongs to the coldspot that included the Futian District and the southern Longhua District (Minzhi Subdistrict). The high–low area on the map which included the Shiyan Subdistrict and the Qingshuihe Subdistrict, indicated that these two subdistricts are high incidence subdistricts compared to other surrounding subdistricts. On the contrary, the Haishan Subdistrict, Longcheng Subdistrict and Kuicong Subdistrict which belongs to a low–high region were the lowest incidence subdistricts compared to other surrounding subdistricts.

## 4. Discussion

Using the temporal trend analysis, a steady increasing trend of lung cancer incidence rate in Shenzhen from 2008 to 2018 was observed in the present study. Although the incidence rate of lung cancer in males was higher than that in females during the study period, the incidence rate of lung cancer in females increased faster than that in males. Among all of the histological types of lung cancer, ADC was the most common, and it showed a continuously increasing trend of incidence. In addition, we also found incidence differences and clustering trends among subdistricts in Shenzhen over the study period, using geographic information system (GIS)-based spatial statistics methods.

To our knowledge, some studies in China have reported the trends of lung cancer incidence in their respective regions. The CR of lung cancer in Beijing from 2000 to 2016 is much higher than that in Shenzhen [7]. However, after the standardization of the incidence rate, the CSR in Beijing is slightly lower than that in Shenzhen, which was indicated that the age composition of Shenzhen residents tends to be younger. Compared with the study of lung cancer incidence collected from the National Central Cancer Registry in China from 2008 to 2012, the age-specific incidence of lung cancer in Shenzhen presented a similar trend. While the CSR and WSR of lung cancer in Shenzhen were slightly higher (37.4/100,000 versus 35.1/100,000 in CSR; 37.5/100,000 versus 34.9/100,000 in WSR) and increased at a faster rate [20]. Therefore, attention still needs to be paid to the prevention and control of lung cancer in Shenzhen. In contrast, in some developed countries, the incidence of lung cancer has shown a decreasing trend due to the continuous decline in smoking rates in recent years [21]. The overall incidence of lung cancer was observed to decline significantly in males and remain stable in females in a U.S. study from 2004 to 2009, which was different from the incidence trend of lung cancer in Shenzhen [22]. In a study from Estonian, the incidence rate of lung cancer declined gradually in males since 1991, but steady increased in females [11]. Differences in lung cancer incidence rates between males and females may be due to smoking habits, genetic susceptibility, biological indicators, hormonal factors, and so on [23,24]. Among them, tobacco smoke may be a main reason of lung cancer. Nevertheless, among females who had much lower smoking rates than males, the reason for the continued increase in lung cancer incidence rate needs to be explored. In some developing countries, such as China, the development of industrialization may be another reason for the increased incidence rate of lung cancer, especially among females. According to a study in Taiwan, the traffic-related NOx and CO have been found to be significantly associated with the incidence of lung cancer in females but not in males [25]. This may be the reason that the annual increase in the incidence rate of lung cancer among females is faster than that among males.

The most common histological type among both sexes in the present study was ADC and the incidence rate of ADC increased continuously throughout the study period, which was the same as the study in Beijing and Sichuan [7,9]. However, the results of a study from Tianjin were not consistent with our [8]. SQCC was the main histological type in male lung cancer patients in Tianjin during 1981 to 2005. In some foreign countries, the histological types of lung cancer have also changed in recent decades, in particular, ADC is more common than SQCC [26,27]. According to relevant studies, changes in lung cancer histological type may be due to improvements in diagnostic techniques or changes in cigarette design and smoking behavior. With advances in diagnostic techniques, it has become easier to perform biopsies on tumors in small, distal airways where ADC often occurs [28]. Not only that, adding filters to cigarettes changes the way smokers smoke, causing them to smoke more intensely into the deeper parts of their lungs where ADC occurs [29]. These changes may be the reason why the proportion of ADC in Shenzhen keeps rising. However, strong cigarette smoke from unfiltered cigarettes can be inhaled shallow, causing the concentration of chemical carcinogens in the bronchial area and causing SQCC [30]. Differences in smoking preferences may partly explain the discrepancy in the histological types of lung cancer among regions. Therefore, changes in the histological type of lung cancer should continue to be of concern, which will contribute to a clearer understanding of the pathogenesis of lung cancer.

By means of the spatial autocorrelation analysis, the present study indicated a non-random distribution of lung cancer incidence in Shenzhen. Among them, the Songgang Subdistrict, Nanao Subdistrict and some subdistricts in the northeast of Shenzhen were at high-risk. Conversely, in some more modernized and higher socioeconomic areas, such as the Nanshan District, the incidence of lung cancer presented a lower risk. It is worth noting that lower socioeconomic status tends to be positively associated with lung cancer rates [31]. In other words, lung cancer rates may be higher in people living in areas of lower socioeconomic level than in areas of higher socioeconomic level. Moreover, people who live in areas of lower socioeconomic levels, especially those with a poorer education, are more likely than those with a higher education or higher socioeconomic levels to be current smokers, and to be physically inactive [32]. Obviously, both smoking and physical inactivity are risk factors for lung cancer, especially smoking. However, while active exercise may help to prevent lung cancer to some extent, it may backfire if you regularly exercise in an air polluted environment. Evidence has been presented showing that air pollution promotes oxidative stress and inflammation in the airways, and exercise may exacerbate these reactions [33]. Therefore, scientific and reasonable exercise and the construction of a healthy living environment are equally important. As urbanization, industrialization and lifestyle diversification move forward, risk exposure levels and disease status vary in different parts of China, as well as Shenzhen. In the past 30 years, the economy in Shenzhen has developed rapidly, from a small fishing village to a highly urbanized city. One of the most worrying consequences of rapid urbanization is stagnant air and the high concentration of associated air pollutants [34]. Notably, the surrounding area in Shenzhen has a higher industrial density than in the central area. Some factories set up in the surrounding areas may also cause more serious environmental pollution than the central area and lead to the occurrence of lung cancer [35]. Studies have confirmed that the air pollution in the northwestern area of Shenzhen is relatively serious, which is consistent with some of our findings [36]. Moreover, Guo et al. have found a strong correlation between PM2.5 and the incidence of male lung cancer in counties (districts) with lower economic levels or lower education levels [37]. In addition, the CR of lung cancer in the surrounding areas of Shenzhen is generally higher than that in the central areas, which may be caused by the differences in population age structure among the districts [38]. The aging degree of the population in the surrounding area is higher than that in the central area, and the proportion of elderly people is higher, which is consistent with the high-risk population of lung cancer [3].

The present study has some limitations. First of all, our study was based on lung cancer incidence data from the cancer registry system. Differences of the medical service, levels of diagnosis among regions may have an impact on the lung cancer incidence. Furthermore, when carrying out spatial autocorrelation regression, we could only use CR for analysis due to the lack of data on populations in different age groups of the subdistrict. The comparison of lung cancer rates in different regions during the study period is likely to be plagued by bias. Therefore, the interpretation of regional clustering results in lung cancer incidence should be very cautious.

## 5. Conclusions

In general, the results of this study are of great significance to public health in Shenzhen. The temporal and spatial analysis of lung cancer incidence not only provides important epidemiological clues for the etiological mechanism of lung cancer, but also provides a scientific basis for the rational allocation of health resources. Future studies should focus on the relationship between spatio-temporal distribution patterns of lung cancer incidence and various risk factors in Shenzhen, so as to better guide the screening and prevention of lung cancer.

## Figures and Tables

**Table 1:** The incidence of lung cancer by sex in Shenzhen, 2008–2018 (1/100,000).

Year	Male				Female				Total			
	N	CR	CSR	WSR	N	CR	CSR	WSR	N	CR	CSR	WSR
2008	348	29.7	45.6	45.5	210	20.4	26.7	26.9	558	25.3	35.9	35.9
2009	371	30.0	44.9	45.3	199	17.9	22.5	22.2	570	24.3	33.3	33.1
2010	443	34.2	52.2	52.1	220	18.8	24.7	24.4	663	26.9	38.0	37.7
2011	412	30.1	44.6	44.9	235	19.2	23.6	23.4	647	24.9	33.9	33.9
2012	457	31.2	44.0	43.2	286	21.8	25.7	25.9	743	26.8	34.6	34.4
2013	468	29.9	42.7	43.2	282	19.8	22.7	22.6	750	25.1	32.2	32.2
2014	493	29.2	44.0	44.1	273	17.9	22.1	21.7	766	23.8	32.5	32.3
2015	578	31.8	49.6	50.5	320	19.8	25.0	24.9	898	26.1	36.9	37.1
2016	613	31.3	49.2	50.7	411	23.6	30.3	30.7	1024	27.7	39.1	39.8
2017	738	34.3	52.6	53.4	527	27.6	34.8	34.6	1265	31.1	43.1	43.2
2018	783	33.7	53.6	54.3	606	29.4	38.0	38.3	1389	31.7	45.3	45.6
Total	5704	31.6	48.0	48.4	3569	22.1	27.7	27.7	9273	27.1	37.4	37.5

N: number of new cases; CR: crude incidence rate; CSR: age-standardized incidence rate according to 2000. Chinese population; WSR: age-standardized incidence rate according to Segi’s world population in 1982.

**Table 2:** The distribution of lung cancer by histological type in Shenzhen, 2008–2018.

Year	Male, N (%)				Female, N (%)				Total, N (%)			
	ADC	SQCC	SMCC	OST	ADC	SQCC	SMCC	OST	ADC	SQCC	SMCC	OST
2008	71(49.7)	43(30.1)	16(11.2)	13(9.1)	66(68.0)	13(13.4)	7(7.2)	11(11.3)	137(57.1)	56(23.3)	23(9.6)	24 (10.0)
2009	91(51.1)	46(25.8)	21(11.8)	20(11.2)	63(68.5)	10(10.9)	9(9.8)	10(10.9)	154(57.0)	56(20.7)	30(11.1)	30(11.1)
2010	111(46.4)	57(23.8)	22(9.2)	49(20.5)	87(71.3)	14(11.5)	3(2.5)	18(14.8)	198(54.8)	71(19.7)	25(6.9)	67(18.6)
2011	114(51.4)	51(23.0)	25(11.3)	32(14.4)	80(73.4)	14(12.8)	3(2.8)	12(11.0)	194(58.6)	65(19.6)	28(8.5)	44(13.3)
2012	156(58.0)	54(20.1)	33(12.3)	26(9.7)	125(74.4)	21(12.5)	5(3.0)	17(10.1)	281(64.3)	75(17.2)	38(8.7)	43(9.8)
2013	147(54.2)	57(21.0)	36(13.3)	31(11.4)	135(77.1)	18(10.3)	4(2.3)	18(10.3)	282(63.2)	75(16.8)	40(9.0)	49(11.0)
2014	197(61.9)	56(17.6)	31(9.7)	34(10.7)	142(80.7)	16(9.1)	5(2.8)	13(7.4)	339(68.6)	72(14.6)	36(7.3)	47(9.5)
2015	216(61.7)	81(23.1)	35(10.0)	18(5.1)	190(86.4)	14(6.4)	2(0.9)	14(6.4)	406(71.2)	95(16.7)	37(6.5)	32(5.6)
2016	226(63.1)	70(19.6)	30(8.4)	32(8.9)	241(88.3)	17(6.2)	5(1.8)	10(3.7)	467(74.0)	87(13.8)	35(5.5)	42(6.7)
2017	333(67.8)	84(17.1)	25(5.1)	49(10.0)	347(91.8)	10(2.6)	7(1.9)	14(3.7)	680(78.3)	94(10.8)	32(3.7)	63(7.2)
2018	361(66.1)	88(16.1)	49(9.0)	48(8.8)	409(89.6)	12(2.6)	10(2.2)	25(5.5)	767(76.8)	100(10.0)	59(5.9)	73(7.3)
Total	2023(59.8)	687(20.3)	352(9.5)	352(10.4)	1882(83.2)	159(7.0)	60(2.7)	162(7.2)	3905(69.1)	846(15.0)	383(6.8)	514(9.1)

ADC: adenocarcinoma; SQCC: squamous carcinoma; SMCC: small cell carcinoma; OST: other specified histological type.

**Table 3:** The trend of age-standardized incidence of lung cancer by histological type in Shenzhen, 2008–2018.

	Year	APC (%)	LCI (%)	UCI (%)
Male				
ADC	2008–2018	10.1 *	8.2	12.1
SQCC	2008–2018	1.1	−1.6	3.8
SMCC	2008–2018	2.6	−2.3	7.7
OST	2008–2018	−0.7	−8.3	7.6
Female				
ADC	2008–2018	14.7 *	11.3	18.1
SQCC	2008–2018	−5.4 *	−10.3	−0.2
SMCC	2008–2018	−2.7	−11.0	6.5
OST	2008–2018	−2.3	−7.9	3.6

ADC: adenocarcinoma; SQCC: squamous carcinoma; SMCC: small cell carcinoma; OST: other specified histological type; APC: annual percent change; LCI: lower limit of the 95% confidence interval; UCI: upper limit of the 95% confidence interval; * *p* < 0.05.

## Data Availability

Not applicable.
